# Seroconversion after influenza vaccination in patients with lung cancer

**DOI:** 10.1038/sj.bjc.6690342

**Published:** 1999-04

**Authors:** H Anderson, K Petrie, C Berrisford, A Charlett, N Thatcher, M Zambon

**Affiliations:** Medical Oncology, Wythenshawe Hospital, Manchester, M23 9LT, UK; University of Manchester Medical School, Department of Surgery, Wythenshawe Hospital, Manchester M23 9LT, UK; PHLS Statistics Unit, 61 Colindale Avenue, London NW9 5HT, UK Respiratory Virus Unit, PHLS Central Public Health Laboratory, 61 Colindale Avenue, London NW9 5HT, UK

**Keywords:** lung cancer, influenza vaccine, antibody response

## Abstract

There are no data in the literature about the efficacy of influenza vaccination in lung cancer patients. Paired sera were available from 59 patients who received Fluvirin (Evans Medical). Of 41 patients susceptible to one or more influenza strains, 78% responded fully to vaccination. This response rate is comparable to that obtained from healthy volunteers. © 1999 Cancer Research Campaign


*British Journal of Cancer* (1999) **80**(1/2), 219–220
© 1999 Cancer Research Campaign
Article no. bjoc.1998.0342

### Seroconversion after influenza vaccination in patients with lung cancer

**H Anderson[1], K Petrie[2], C Berrisford[1], A Charlett[3], N Thatcher[1] and M Zambon[4]**

[1]Medical Oncology, Wythenshawe Hospital, Manchester M23 9LT, UK; [2]University of Manchester Medical School, Department of Surgery, Wythenshawe Hospital, Manchester M23 9LT, UK; [3]PHLS Statistics Unit, 61 Colindale Avenue, London NW9 5HT, UK; [4]Respiratory Virus Unit, PHLS Central Public Health Laboratory, 61 Colindale Avenue, London NW9 5HT, UK

**Summary** There are no data in the literature about the efficacy of influenza vaccination in lung cancer patients. Paired sera were available from 59 patients who received Fluvirin (Evans Medical). Of 41 patients susceptible to one or more influenza strains, 78% responded fully to vaccination. This response rate is comparable to that obtained from healthy volunteers.

**Keywords:** lung cancer; influenza vaccine; antibody response

The Department of Health recommends annual vaccination for patients at high risk of morbidity and mortality from influenza (Department of Health, 1996). Patients with lung cancer fall into this category because of chronic respiratory disease and immunosuppression due to cancer or treatment. There are increasing data on the impact of influenza in adults with cancer (Yousuf et al, 1997), but knowledge of serological responses to inactivated flu vaccine in adults with cancer, particularly those treated with steroids, remains scanty.

### PATIENTS AND METHODS

Between October and November 1996, patients with lung cancer attending the Northwest regional oncology outpatient clinic were asked to participate in a study to assess serological responses to inactivated influenza vaccine. Local ethics approval was obtained and patients gave written informed consent. Individuals were excluded from the study if they had already received vaccine from their GP or were allergic to eggs. Fluvirin (Evans Medical Ltd) containing inactivated A/Singapore/6/86 (H1N1), A/Wuhan/359/95 (H3N2) and B/Beijing/184/93 was administered as a single subcutaneous injection. A 10 ml serum sample was taken prior to vaccination and a second sample 4–6 weeks later. Prevaccination susceptibility and post-vaccination serological responses to the three influenza antigens were determined by haemagglutination inhibition (HI). A reciprocal HI titre of ≥ 40 was considered protective. All patients were given a viral swab kit and instructions on collecting nose and throat swabs if they developed symptoms suggestive of influenza.

*Received 23 April 1998*
*Revised 28 August 1998*
*Accepted 13 October 1998*

*Correspondence to:* H Anderson, Chest Clinic, North-West Lung Centre, Wythenshawe Hospital, Southmoor Road, Wythenshawe M23 9LT, UK

### RESULTS

Sixty-seven patients were recruited, of which 59 (36 male) had paired sera available for analysis. The median age was 62 years (range 45–75). Twenty one had small cell (14 extensive stage) and 38 non-small cell lung cancer (35 TNM stage III–IV disease). Twenty patients received influenza vaccine in the previous year. Fourteen patients had received chemotherapy in the preceding 4 weeks and 22 patients were receiving oral corticosteroids.

Prevaccination HI serology showed that 18/59 patients (31%) were immune to all three influenza strains. Twelve of these (67%) were individuals who had received influenza vaccine the previous year. 21/59 patients (36%) were susceptible to all three virus strains, and the remaining 34% (20/59) were susceptible to either one or two strains of influenza. 62% (13/21) of fully-susceptible individuals made a complete protective response to influenza vaccine and a further seven (33%) made a protective response to one or two antigens (Table 1). Only one fully-susceptible patient failed to make any protective responses. Overall, 83% of all patients were fully immune to influenza following vaccination. During the course of the study, five patients submitted combined

**Table 1** Pre- and post-vaccination influenza virus susceptibility

| Prevaccination status | *n* | Post-vaccination protection* (*n* (%)) | | |
|---|---|---|---|---|
| | | Full | Partial | None |
| Immune to all 3 strains | 18 | 17 (94%) | 1 (6%) | 0 |
| Susceptible to | | | | |
| 3 strains | 21 | 13 (62%) | 7 (33%) | 1 (5%) |
| 2 strains | 11 | 10 (91%) | 1 (9%) | 0 |
| 1 strain | 9 | 9 (100%) | 0 | 0 |
| Total | 41 | 32 (78%) | 8 (20%) | 1 (2%) |
| Grand Total | | 49 (83%) | | |

*Full protection is defined as HI titre ≥ 40 to all three influenza antigens; partial protection is defined as HI titre ≥ 40 to one or two influenza antigens.

Fluvirin supplied by Evans Medical

**Table 2** Post-vaccination influenza virus protection analysed according to patient variables

| Subgroup (*n*) | Immune prevaccination (*n* (%)) | Post-vaccination protection (*n* (%)) | | |
|---|---|---|---|---|
| | | Full | Partial | None |
| Small cell histology (38) | 11 (29%) | 32 (84%) | 5 (13%) | 1 (3%) |
| Non-small cell histology (21) | 7 (33%) | 17 (81%) | 4 (19%) | 0 |
| Recent chemotherapy (14) | 3 (21%) | 11 (79%) | 3 (21%) | 0 |
| No chemotherapy (45) | 15 (33%) | 38 (84%) | 6 (13%) | 1 (2%) |
| On steroids (22) | 7 (32%) | 19 (86%) | 3 (14%) | 0 |
| No steroids (37) | 11 (30%) | 30 (81%) | 6 (16%) | 1 |

*P* values for small cell vs non-small cell histology, chemotherapy vs no recent chemotherapy and systemic corticosteroids vs none were all > 0.6 (Fishers exact test).

nose and throat swabs for virus culture, from which no viruses were recovered.

Analysis of factors which may have influenced vaccine response in those susceptible (Table 2), using a comparison of independent proportions ($\chi^2$ test), showed that lung cancer histology, chemotherapy in the previous 4 weeks or systemic steroid medication had no effect on seroconversion, or the attainment of a protective HI response in susceptible lung cancer patients. A within-subject linear model was used to estimate the increase in HI titre post-vaccination; this was 13-fold for H1N1, 7-fold for H3N2, and 6-fold for influenza B. There was weak evidence that the increase in HI titre post-vaccination was larger as patient white cell counts increased for both A strains ($P$ = 0.06 for H1N1, $P$ = 0.08 for H3N2), but not for the B strain.

### DISCUSSION

The serological responses obtained from this group of lung cancer patients indicates that 32/41 (78%) of lung cancer patients susceptible to one or more influenza strains responded fully to vaccination with inactivated influenza vaccine. This level of response is comparable to responses obtained from normal healthy volunteers (Department of Health, 1996; Lorio et al, 1989).

There are several studies of influenza vaccination in malignancy. Shildt et al (1979) found that lymphoma patients had the lowest antibody responses. He looked at 82 patients with different malignancies, only 14 had lung cancer. Ortbals et al (1977) studied 42 patients who received whole virus vaccine of whom 21 had solid tumours and one had lung cancer. The most interesting result of this study was data showing a 50% response rate if patients were vaccinated at the time of chemotherapy but a 93% response rate if they were vaccinated between courses of chemotherapy. In our study, all patients on chemotherapy were vaccinated between courses of treatment.

Research into new therapies for lung cancer includes tumour vaccine therapy. The ability of lung cancer patients to develop a protective response to influenza vaccine despite chemotherapy and systemic corticosteroids suggests that this approach may be useful.

In this small study, protective responses to influenza vaccine did not appear to be affected by systemic steroid treatment, recent chemotherapy or lung cancer histology.

Less than 50% of high-risk patients receive influenza vaccine for a variety of reasons (Editorial, 1997). Advice from treating physicians to general practitioners that influenza vaccine is effective in lung cancer patients may increase its use (Watkins, 1997).

### ACKNOWLEDGEMENTS

The authors thank Evans Medical for provision of inactivated vaccine and Paul Laidler for excellent technical assistance.

### REFERENCES

Department of Health (1996) *Immunisation against Infectious Disease*, Salisbury DM, Begg NT, (eds) pp 113–120. HMSO: London

Editorial (1997) Uptake of vaccine in high risk patients. *Commun Dis Rep Weekly* **7**: 407

Lorio AM, Rivosecchi P, Zei P, Neri M and Merlett L (1989) Immune response to trivalent inactivated influenza vaccine in young and elderly subjects. *Vaccine* **7**: 303–306

Ortbals DW, Liebhaber H, Presant CA, van Amburg AL and Lee JY (1977) Influenza immunisation of adult patients with malignant diseases. *Ann Intern Med* **87**: 552–557

Shildt RA, Luedke DW, Kasai G, El-Beheri S and Laham MN (1979) Antibody response to influenza immunisation in adult patients with malignant disease. *Cancer* **44**: 1629–1635

Watkins J (1997) Effectiveness of influenza vaccination policy at targeting patients at high risk of complications during winter 1994–95: Cross Sectional Survey. *BMJ* **315**: 1069–1070

Yousuf HM, Englund J, Couch R, Rolston K, Luna M and Goodrich J (1997) Influenza among hospitalised adults with leukaemia. *Clinical Infectious Diseases* **24**: 1095–1099

 *© Cancer Research Campaign 1999*
